# Characterizing Disease Modifying Effects Using Overall Treatment Effect Across all Post‐Baseline Visits

**DOI:** 10.1002/alz.087728

**Published:** 2025-01-09

**Authors:** John O'Gorman, Tianle Chen, Guoqiao Wang

**Affiliations:** ^1^ Biogen, Cambridge, MA USA; ^2^ Washington University School of Medicine, St. Louis, MO USA

## Abstract

**Background:**

The well‐accepted statistical efficacy inference approach for Alzheimer’s disease (AD) clinical trials compares the absolute difference in change from baseline at the last study visit using MMRM (henceforth referred to as MMRM‐Last‐Visit). Recent AD clinical trials have shown that treatment effects may be manifested prior to 18 months. The objective is to evaluate models estimating an overall treatment effect across all post‐baseline visits that may characterize disease modifying effects in contemporary early AD clinical trials.

**Method:**

We will evaluate the performance of: (1) MMRM‐Last‐Visit, (2) MMRM‐All‐Visits, estimating an overall treatment effect across all post‐baseline visits, (3) Linear mixed effects (LME) model with time being continuous, comparing the slopes across treatment groups, (4) Constrained LME, comparing the slopes which constraining the treatment groups to have the same mean baseline, (5) Longitudinal data analysis model with natural cubic spline in time (LDA‐NCS), modeling the raw data and comparing the absolute difference in change from baseline at the last visit (LDA‐NCS ‐Last‐Visit), (6) Constrained LDA‐NCS, similar to LDA‐NCS while constraining treatment groups to have the same mean baseline, and (7) proportional/percentage MMRM (pMMRM), which models the percentage reduction across all post‐baseline visits. Simulated data mimicking disease modifying effect in contemporary early AD trials were used for the evaluation. Subsequently, we evaluate the performance using bootstrapping techniques.

**Result:**

We will present simulation results that mimic a range of assumptions from contemporary early AD trials, including sample size, power, and type I error results.

**Conclusion:**

Models estimating an overall treatment effect across all post‐baseline visits have shown promise in characterizing disease modifying effects seen in contemporary early AD clinical trials and may help accelerating drug development.

This study was funded by Biogen, JO and TC are employees and shareholders of BIogen.

